# Concomitant Effects of Ramadan Fasting and Time-Of-Day on Apolipoprotein AI, B, Lp-a and Homocysteine Responses during Aerobic Exercise in Tunisian Soccer Players

**DOI:** 10.1371/journal.pone.0079873

**Published:** 2013-11-11

**Authors:** Omar Hammouda, Hamdi Chtourou, Asma Aloui, Henda Chahed, Choumous Kallel, Abdelhedi Miled, Karim Chamari, Anis Chaouachi, Nizar Souissi

**Affiliations:** 1 Research Laboratory “Sport Performance Optimization”, National Center of Medicine and Sciences in Sport (CNMSS), Tunis, Tunisia; 2 Research Laboratory of Biochemistry, CHU Farhat Hached, Sousse, Tunisia; 3 Research Laboratory of Hematology, CHU Habib Bourguiba, Sfax, Tunisia; 4 High Institute of Sport and Physical Education of Ksar-Saïd, Manouba University, Manouba, Tunisia; 5 High Institute of Sport and Physical Education of Sfax, Sfax University, Sfax, Tunisia; 6 Research and Education Center, Aspetar, Qatar Orthopedic and Sports Medicine Hospital, Doha, Qatar; Azienda Policlinico S. Orsola-Malpighi, Italy

## Abstract

**Objective:**

To examine the time-of-day and Ramadan fasting (RF) effects on serum apolipoprotein-AI (Apo-AI) and B (Apo-B), lipoprotein particles-a (Lp-a), high-sensitive C-reactive-protein (hs-CRP), and homocysteine (Hcy) during the Yo-Yo intermittent recovery test (YYIRT).

**Design:**

Performance and biochemical measures were completed at two times-of-day (07:00 and 17:00 h), 1-week before RF (BR), the second week of RF (SWR), and the fourth week of RF (ER).

**Setting:**

For each session, subjects performed the YYIRT, and blood samples were taken before and 3-min after the test for biochemical measures.

**Participants:**

Fifteen soccer players.

**Main Outcome Measures:**

Total distance during the YYIRT, core temperature, body composition, dietary intakes, lipid (HDL-C, LDL-C, Apo-AI, B and Lp-a) and inflammatory (hs-CRP and Hcy) profiles.

**Results:**

Performances during the YYIRT were higher in the evening than the morning BR (*P* < 0.05), but this fluctuation was not observed during RF. Moreover, LDL-C, ApoB, and Lp-a were stable throughout the daytime BR. However, during RF, they decreased at 17:00 h (*P* < 0.05). Likewise, HDL-C and Apo-AI increased after the exercise and were higher at 17:00 h BR (*P* < 0.001). Moreover, these parameters increased during RF (*P* < 0.01). Furthermore, Hcy and hs-CRP increased during the exercise (*P* < 0.01) with higher evening levels BR. During ER, the diurnal pattern of Hcy was inversed (*P* < 0.001).

**Conclusions:**

This study concluded that caloric restriction induced by RF seems to ameliorate lipid and inflammatory markers of cardiovascular health during intermittent exercise performed in the evening.

## Introduction

The Ramadan fast (RF) is observed by Muslims every year when they are required to abstain from food and fluid intake between dawn and dusk. It is well established that RF induces many beneficial effects on lipid metabolism in healthy subjects [1-4]. Indeed, it has been demonstrated that RF is accompanied with reduced serum low density lipoprotein (LDL-C), apolipoprotein B (Apo-B), a major protein component of LDL-C, total cholesterol (TC), triglycerides (TG), and increased apolipoprotein AI (Apo-AI) and high density lipoprotein (HDL-C) levels, with unchanged or reduced body mass despite, in some cases, increased caloric intake [1,3,5]. In this context, Apo B, as a major protein component of LDL-C, has been proposed to be a better indicator for coronary artery diseases than TC and TG [6]. Moreover, Apo-AI-containing lipoprotein particles (Lp-a) seem to be a more powerful discriminating marker for cardiovascular risk than Apo-AI and HDL-C [7]. However, several other studies have found a decline in HDL-C levels and an increase in LDL-C levels following Ramadan fasting [8,9]. 

In the study of Adlouni et al [3], Apo-AI increased in the day 8 and 29 of RF and remained elevated one month later in comparison with before RF. However, Apo-B levels were lower during the second and the last week of RF and remained low one month after RF. Moreover, it has been reported that RF positively influenced the inflammatory status by a decrease in the pro-inflammatory cytokine (ie, IL-6) concentrations associated with decreases in both high-sensitive C-reactive protein (hs-CRP) and the cardiovascular-risk marker (ie, homocysteine (Hcy)) [10]. Nevertheless, the studies cited above were conducted in healthy sedentary subjects. In highly trained subjects, very few studies investigated the influence of RF on lipid metabolism. Chaouachi et al [11] reported changes in some serum lipids and apolipoproteins (ie, increase in HDL-C, LDL-C, and Apo-AI) in elite judo athletes. Conversely, only free-fatty-acid and HDL-C levels have been shown to increase during RF in middle-distance runners [1,3,5] and physically active men [13]. Particularly, it has recently been identified that aerobic training during RF may increase muscle oxidative capacity [14] and enhance exercise-induced intramyocellular lipid degradation [15]. It seems that the effect of RF on serum lipid levels may be closely related to the feeding behavior or biochemical responses to starvation [3]. Concerning the RF effect on exercise, the few studies conducted in this context indicated an increase in plasma TG and HDL-C concentrations during submaximal exercise [16,17].

Otherwise, most clinical biochemical entities have been shown to exhibit circadian rhythms at rest [18] and during exercise [19,20] in soccer players. In this context, it has been indicated that fasting and postprandial lipid, lipoprotein, and apolipoprotein concentrations were affected by circadian factors [21,22]. Moreover, it has been established that a given nutrient ingested at an unusual time could induce different metabolic effects [23]. In fact, our previous findings indicated that the resting circadian pattern of lipid metabolism could influence post-exercise measurements [24]. Furthermore, we recently observed that various components of physical performances displayed diurnal variations (ie, higher evening values) which tend to disappear during RF [25]. Even if the circadian rhythm in core temperature is not necessarily the cause of the rhythm in muscle performance [26], previous research has suggested that the simultaneous increases in body temperature and muscular power are causally related. Moreover, it is well known that RF modifies the diurnal pattern of core temperature, which could affect the diurnal patterns of biochemical measures [27].

In view of the above considerations, the modified biochemical response during RF could be a consequence of various external factors. The objective of the present study was to examine the RF effects on daily fluctuations in serum lipoproteins, Apo-AI, Apo-B, Lp-a as well as hs-CRP and Hcy responses during a maximal aerobic exercise in soccer players. The selected markers are important indicators of cardiovascular health and post exercise measurements during RF. Therefore, it would be of interest to investigate the modified metabolic response to exercise. We hypothesize that RF influences the lipid profile and inflammatory status through the concomitant effects of many mechanisms (ie, diet, time-of-day, week of Ramadan).

## Methods

### Subjects

Fifteen male professional soccer players (age, 17.3 ± 0.3 years, body mass, 69.1 ± 4.2 kg, height, 179.7 ± 3.6 cm) volunteered to participate in this study. The participants were recruited on the basis of: (i) they trained in the evening at least 4 days per week for an average of 2 h daily in addition to the weekend match and (ii) they had a minimum of 5 years of training experience and were members of the same youth team competing in the first division of the Tunisian football league. The training program during RF consisted of high intensity short duration intermittent exercises. We interviewed all players and coaches in order to provide information concerning the number of years of football practice and hours of regular training per week. Moreover, goalkeepers and players who experienced injuries were excluded from the analysis. So, the volunteers were homogeneous in physical characteristics (ie, VO2max, strength, anaerobic power and capacity). Approval for the study was obtained from the club. Subjects abstained from food, drinks and sexual activity about 16 h/day during Ramadan. The last meal was taken at 1:00–2:00 h. The study was carried out in Sfax, Tunisia in 2010 when Ramadan started on the 11^th^ of August 2010 and ended on the 10^th^ of September 2010. The length of each fasting day was approximately 15-16 h. To ensure that participants were all of a “moderately morning type” (n = 5) or “neither type” (n = 10), they were selected based on their responses to the Horne and Östberg’s self-assessment questionnaire [28]. The Horne and Östberg’s questionnaire allowed selecting subjects of similar sleeping and daily habits which allows a better understanding of the modified diurnal pattern of biochemical responses. Subjects were nonsmokers and did not consume nutritional supplements, caffeine, drugs, or alcoholic beverages.

### Experimental Design

The experimental design consisted of three testing periods: (i) one week before RF (BR), the second week of RF (SWR), and the fourth week of RF (ER). For example, the major change of HDL-C and LDL-C levels were observed during the first and the last weeks of RF [29], that’s why we chose to evaluate our subjects in the second and the fourth weeks of Ramadan. At each phase, the subjects performed two test sessions (TS) at two times-of-day (ie, 07:00 and 17:00 h) with a recovery period ≥ 36 h in-between. These two time points were chosen because they correspond to the peak and trough of football-specific skills and measures of physical performance [30]. TS were completed in a counterbalanced design and each one started with measure of oral temperature with a digital clinical thermometer (Omron, Paris, France; accuracy ± 0.05°C) inserted sublingually for at least 3 min after a 10-min rest in a seated position. To ensure a reliable measure of temperature, subjects were instructed not to talk or drink during measurement. Then, the soccer players participated in an endurance specific test (the Yo-Yo intermittent recovery test level-1 (YYIRT)). Heart rate was recorded during the YYIRT using a Polar heart rate monitor (Polar Electro Oy, T61-coded, Finland).

Body composition was assessed using an electronic scale (Tanita, Tokyo, Japan) during each testing phase [19]. Throughout the experimental period, participants were required to maintain their habitual physical activity and to avoid strenuous physical efforts 24 h before each TS.

### The Yo-Yo Intermittent Recovery Test Level-1 (YYIRT)

As described previously [24], the test consisted of 20-m shuttle runs performed at increasing velocities with 10 s of active recovery between runs until exhaustion. Moreover, the reliability of the YYIRT was established in a previous study [31]. The end of the test was considered when the participant twice failed to reach the front line in time or he felt unable to complete another shuttle at the dictated speed. The total distance covered during the YYIRT (including the last incomplete shuttle) was considered as the test score. All players were already familiar with the test as it was part of their usual fitness assessment program. We have chosen the YYIRT due to its specificity to soccer. Indeed, Castagna et al [31] reported a significant correlation (r = 0.65) between the total distance covered during the YYIRT and the total distance covered during a soccer match. 

### Dietary Records

A seven consecutive day dietary record was completed. Dietary records were done in the same weeks as TS. Subjects received a detailed verbal explanation and written instructions on data collection procedures. They were asked to be as accurate as possible in recording the amount and type of food and fluid consumed. Each individual’s diet was calculated using the Bilnut 4 software package (SCDA Nutrisoft, Cerelles, France) and the food composition tables published by the Tunisian National Institute of Statistics in 1978 (Table 1). To additionally substantiate the effect of acute exercise on the estimation of Hcy, Folate and vitamin B12 intakes were estimated from the subjects’ dietary intake using a self-administrated quantitative food questionnaire [32].

**Table 1:** Body composition and dietary intakes before Ramadan (BR), during the second week of Ramadan (SWR), and during the fourth week of Ramadan (ER).

	**BR**	**SWR**	**ER**
**Body mass (kg)**	67.22 ± 16.2	65.90 ± 14.8*	64.80 ± 14.9*
**Fat mass (%)**	16.20 ± 7.1	16.01 ± 6.9	15.60 ± 5.5*
**Lean mass (kg)**	61.20 ± 6.4	60.90 ± 6	60.50 ± 6.2
**Daily energy intake (Kcal)**	3302 ± 709.8	2900.91 ± 738*	2693 ± 517.13*
**Carbohydrates (g)**	422.12 ± 144.81	398.26 ± 96.13	415.35 ± 94.41
**Protein (g)**	96.86 ± 21.01	98.72 ± 37.88	96.38 ± 30.6
**Fat (g)**	110.61 ± 63.26	105.64 ± 38.40	101.29 ± 38.18*
**Cholesterol (mg.d^-1^)**	357.21 ± 224.17	525.06 ± 283.40*	461.48 ± 334.14
**Saturated fat (g)**	33.04 ± 8.84	27.19 ± 7.45*	26.85 ± 4.72*
**Monounsaturated fat (g)**	40.33 ± 5.58	38.39 ± 7.66	40.28 ± 6.64
**Polyunsaturated fat (g)**	26.5 ± 9.28	34.91 ± 9.10*	30.97 ± 9.93
**Vitamin C (mg.d^-1^)**	46.64 ± 30.92	44.73 ± 41.97	43.09 ± 57.92
**Vitamin E (mg.d^-1^)**	4.05 ± 2.11	5.19 ± 3.60	4.39 ± 1.44
**Vitamin A (ER)**	2115.3 ± 1.51	1805.3 ± 1.41	1708.4 ± 1.45
**Vitamin B12 (µg. d^-1^)**	7.3 ± 1.5	7.4 ± 1.7	7.2 ± 0.9
**Folate (µg. d^-1^)**	340.96 ± 58	342.34 ± 46	343.57 ± 63

Values are expressed as mean ± SD.

* Significantly different from pre-Ramadan value.

### Blood Sample Variables Analysis

Blood samples were obtained from a forearm vein after 5 min of seated rest and 3 min after the YYIRT. TC, TG, and HDL-C were estimated by standard enzymatic analysis using reagents, standards, and controls from Randox Laboratories Ltd. (Antrim, UK). The coefficients of variation (CVs) for these parameters were < 7%. LDL-C was calculated by the Friedewald formula for TG levels below 400 mg/dL [24]. The above measures were done as adapted for the autoanalyzer by Synchron CX systems (Beckman Instruments, Danville, California, USA).

Determination of Apo-AI, Apo-B, Lp-a, and hs-CRP were performed by rate nephelometry on an immunonephelometer analyzer (BN Prospec, Siemens Diagnostics) using mono- and polyclonal antibodies. The inter- and intra-assay CVs for these parameters were < 2.8% and < 4.7% respectively. Hcy was determined using immunoassay IMX Analyzer (Abbott Laboratories, Lake Bluff, Illinois, USA). The detection limit of the assay was 0.8 μmol·L^-1^ and the intra- and inter-assay imprecision CVs were 2.3% and 3.2% respectively. Plasma volume changes were estimated based on resting and post-exercise haematocrit and hemoglobin using the equations of Dill and Costill [33]. Haematocrit was measured by micro-centrifugation and hemoglobin was assessed by the cyanmethemoglobin method.

### Statistical Analysis

All statistical tests were processed using STATISTICA Software (StatSoft, France). The Shapiro-Wilk *W*-test of normality revealed that the data were normally distributed. The data were analyzed using a two-way analysis of variance (ANOVA) [3 (Ramadan) × 2 (time-of-day)] for the YYIRT, oral temperature, and peak heart rate (HRpeak), and a three-way ANOVA [3 (Ramadan) × 2 (time-of-day) × 2 (blood samples)] with repeated measures for the biochemical measurements. When appropriate, significant differences between means were tested using the Tukey post-hoc test. Paired t-tests were used to compare the morning-evening differences between BR, SWR, and ER. A probability level of 0.05 was selected as the criterion for statistical significance.

### Ethical Considerations

After receiving a description of the protocol and its benefits and possible risks, each volunteer (and parents/tutors for the minors) provided a written informed consent. The experimental design was approved by the Clinical Research Ethics Committee of the National Center of Medicine and Sciences in Sport of Tunis and met the ethical standards of the Declaration of Helsinki.

## Results

### Body Composition and Dietary Intake

Body mass (BM), fat mass (FM), and lean mass data are shown in Table 1. Statistical analyses indicated a decrease in BM and FM by the ER with respect to BR. Nevertheless, lean mass was unaffected by RF. Likewise, there was no significant difference between SWR and ER.

Compared with BR, the estimated daily energy intake was substantially reduced by the ER. This change was associated with a decrease in fat and saturated fat intakes and an increase in polyunsaturated fat, while carbohydrates and protein intakes remained stable during RF. Moreover, Folate, Vitamin A, Vitamin B12, Vitamin C, and Vitamin E intakes were unchanged during RF. For all these parameters, there was no significant difference between SWR and ER.

### Temperature and YYIRT Performance

**Figure 1:**
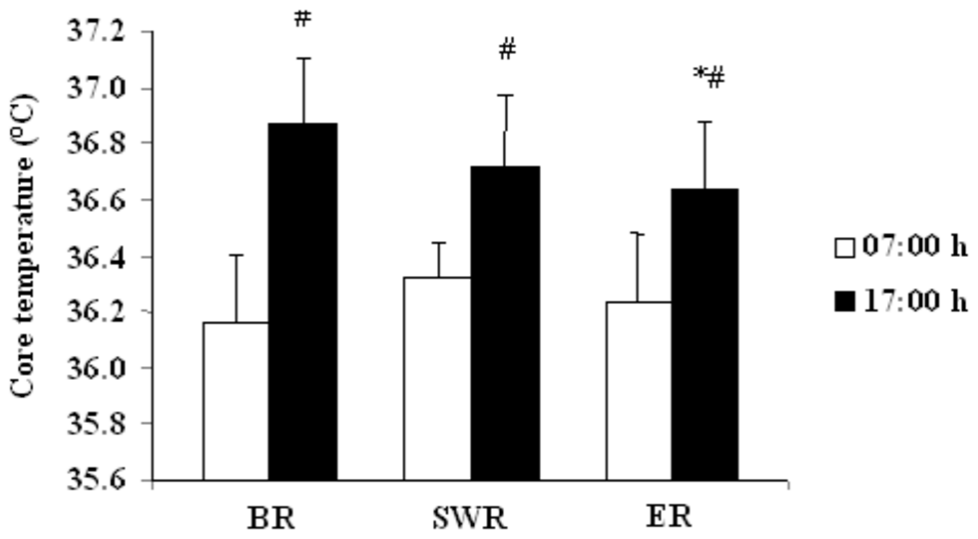
Oral temperature (°C) measured at 07:00 h and 17:00 h before Ramadan (BR), during the second week of Ramadan (SWR), and during the fourth week of Ramadan (ER). Values are expressed as mean ± SD. *Significantly different from pre-Ramadan value for the same time-of-day. ^#^Significantly different from 07:00 h value for the same period.

Oral temperature increased significantly from the morning to the evening BR, and during SWR and ER (*P* < 0.001). This increase was greater BR than SWR (+0.71 ± 0.32°C *vs.* +0.4 ± 0.28°C; t = 2.3; *P* < 0.05) and ER (+0.71 ± 0.32°C *vs.* +0.41 ± 0.32°C; t = 2.1; *P* < 0.05) (Figure 1). However, there was no significant difference between SWR and ER (+0.4 ± 0.28°C *vs.* +0.41 ± 0.32°C; t = 0.07). During RF, in comparison with BR, oral temperature was not affected in the morning. However, there was a significant decrease in oral temperature at ER in the evening.

The results showed that performance during the YYIRT improved between the morning and the evening BR (*P* < 0.05). However, these diurnal variations were not found during SWR and ER (Table 2) due to a significant decrease in morning-evening differences between BR and SWR (280 ± 237.32 *vs.* 36.36 ± 265.6; t = 2.3; *P* < 0.05) and between BR and ER (280 ± 237.32 *vs.* 101.82 ± 415.06; t = 2.6; *P* < 0.05). There was no significant difference between SWR and ER (36.36 ± 265.6 *vs.* 101.82 ± 415.06; t = 0.9). Moreover, performance during the YYIRT decreased significantly during SWR and ER (*P* < 0.01 and *P* < 0.001 respectively) only at 17:00 h. Furthermore, the results revealed that HRpeak decreased during SWR and ER in the evening (*P* < 0.05, Table 2).

**Table 2:** TD and HRpeak measured at the two times-of-day before Ramadan (BR), during the second week of Ramadan (SWR), and during the fourth week of Ramadan (ER).

	**BR**		**SWR**		**ER**	
	**07:00 h**	**17:00 h**	**07:00 h**	**17:00 h**	**07:00 h**	**17:00 h**
**TD (m)**	1763.64 ± 482.48^#^	2043.64 ± 533.5	1720 ± 626.87	1756.36 ± 503.57*	1727.27 ± 590.27	1625.45 ± 562.07*
**HRpeak (beats · min^-^1)**	190.9 ± 5.1	192 ± 7.3	190.8 ± 3.7	188 ± 4.8*	188.9 ± 2.4	187.9 ± 4.3*

Values are expressed as mean ± SD.

* Significantly different from pre-Ramadan value for the same time-of-day.

^#^ Significantly different from 17:00 h value for the same period.

YYIRT: the Yo-Yo intermittent recovery test; TD: total distance covered during the YYIRT; HRpeak: peak heart rate during the YYIRT.

### Biochemical Measurements

Before RF, TC and TG levels increased significantly during the YYIRT and showed a significant diurnal variation with higher levels at 17:00 h compared to 07:00 h (*P* < 0.01). However, during the SWR, although the values were similar to BR at 07:00 h, they decreased in the evening to reach a value indicative of blunted diurnal variations. At the ER, morning and evening levels of TC and TG decreased significantly (*P* < 0.01) in comparison with BR (Table 3).

**Table 3:** Resting and post-exercise biochemical measurements in the morning and the evening before Ramadan (BR), during the second week of Ramadan (SWR), and during the fourth week of Ramadan (ER).

		**BR**		**SWR**		**ER**		**Ramadan effect**	**Time-of-day effect**	**Blood samples effect**	**Ramadan × Time-of-day**	**Ramadan × Blood samples**	**Time-of-day × Blood samples**
		**Morning**	**Evening**	**Morning**	**Evening**	**Morning**	**Evening**						
**TC (mmol·L^-1^)**	Rest	3.06 ± 0.80^#^	3.39 ± 0.72	3.00 ± 0.34^#^	3.27 ± 0.20	2.99 ± 0.21	3.08 ± 0.45*	F = 0.73; p>0.05	F = 18.92; p<0.05	F = 23.08; p<0.05	F = 1.37; p>0.05	F = 0.13; p>0.05	F = 0.06; p>0.05
	Post-exercise	3.34 ± 0.59^#$^	3.67 ± 0.56^$^	3.35 ± 0.31^#$^	3.61 ± 0.42^$^	3.28 ± 0.24^$^	3.36 ± 0.38* ^$^						
**TG (mmol·L^-1^)**	Rest	1.01 ± 0.29^#^	1.20 ± 0.26	0.98 ± 0.22	1.06 ± 0.26*	0.96 ± 0.22	1.00 ± 0.25*	F = 7.98; p<0.05	F = 30.88; p<0.05	F = 25.27; p<0.05	F = 8.31; p<0.05	F = 3.28; p>0.05	F = 1.74; p>0.05
	Post-exercise	1.21 ± 0.46^#$^	1.56 ± 0.54^$^	1.13 ± 0.22^$^	1.22 ± 0.25* ^$^	1.06 ± 0.17*	1.11 ± 0.17*						
**HDL-C (mmol·L^-1^)**	Rest	1.06 ± 0.14^#^	1.18 ± 0.19	1.08 ± 0.19^#^	1.14 ± 0.17	1.16 ± 0.06*	1.20 ± 0.10	F = 3.57; p<0.05	F = 48.84; p<0.05	F = 36.87; p<0.05	F = 2.75; p>0.05	F = 0.31; p>0.05	F = 0.03; p>0.05
	Post-exercise	1.22 ± 0.06^#$^	1.34 ± 0.12^$^	1.27 ± 0.13* ^#$^	1.32 ± 0.10^$^	1.36 ± 0.16* ^$^	1.41 ± 0.15* ^$^						
**LDL-C (mmol·L^-1^)**	Rest	1.33 ± 0.21	1.32 ± 0.13	1.30 ± 0.15^#^	1.23 ± 0.14*	1.26 ± 0.13* ^#^	1.13 ± 0.07*	F = 11.39; p<0.05	F = 11.69; p<0.05	F = 25.59; p<0.05	F = 4.01; p<0.05	F = 1.15; p>0.05	F = 1.72; p>0.05
	Post-exercise	1.39 ± 0.20^$^	1.38 ± 0.14^$^	1.37 ± 0.16^#$^	1.29 ± 0.16* ^$^	1.32 ± 0.09* ^#$^	1.25 ± 0.07* ^$^						
**Apo-AI (g.L^-1^)**	Rest	1.32 ± 0.16^#^	1.44 ± 0.19	1.34 ± 0.10^$^	1.42 ± 0.06	1.40 ± 0.09*	1.42 ± 0.12	F = 0.14; p>0.05	F = 18.04; p<0.05	F = 32.62; p<0.05	F = 4.73; p<0.05	F = 0.14; p>0.05	F = 0.91; p>0.05
	Post-exercise	1.45 ± 0.25 ^#$^	1.63 ± 0.25^$^	1.47 ± 0.15^#$^	1.60 ± 0.19^$^	1.54 ± 0.12* ^$^	1.54 ± 0.10* ^$^						
**Apo-B (g.L^-1^)**	Rest	0.55 ± 0.12	0.56 ± 0.11	0.53 ± 0.11*	0.55 ± 0.12	0.51 ± 0.06	0.50 ± 0.08*	F = 5.49; p<0.05	F = 0.74; p>0.05	F = 34.26; p<0.05	F = 1.06; p>0.05	F = 1.7; p>0.05	F = 0.1; p>0.05
	Post-exercise	0.62 ± 0.11^$^	0.64 ± 0.11^$^	0.61 ± 0.12^$^	0.62 ± 0.11^$^	0.55 ± 0.04*	0.55 ± 0.07* ^$^						
**Lp-a (g.L^-1^)**	Rest	0.15 ± 0.09	0.13 ± 0.05	0.14 ± 0.08	0.15 ± 0.08	0.14 ± 0.03	0.10 ± 0.01*	F = 1.67; p>0.05	F = 5.61; p<0.05	F = 0.58; p>0.05	F = 1.09; p>0.05	F = 0.02; p>0.05	F = 0.02; p>0.05
	Post-exercise	0.16 ± 0.06	0.14 ± 0.03	0.15 ± 0.08	0.14 ± 0.04	0.15 ± 0.03	0.11 ± 0.02						
**hs-CRP (mg.L^-1^)**	Rest	0.72 ± 0.20	0.84 ± 0.16	0.71 ± 0.34	0.68 ± 0.39*	0.73 ± 0.32	0.60 ± 0.30*	F = 1.24; p>0.05	F = 0.05; p>0.05	F = 14.86; p<0.05	F = 4.33; p<0.05	F = 0.78; p>0.05	F = 0.9; p>0.05
	Post-exercise	0.90 ± 0.36^#$^	1.04 ± 0.42^$^	0.86 ± 0.39^$^	0.81 ± 0.44* ^$^	0.84 ± 0.33	0.71 ± 0.29*						
**Hcy (μmol·L^-1^)**	Rest	15.82 ± 0.87^#^	16.82 ± 0.77	15.39 ± 0.67*	15.57 ± 0.65*	14.83 ± 0.64* ^#^	14.40 ± 0.63*	F = 39.63; p<0.05	F = 39.21; p<0.05	F = 47.67; p<0.05	F = 68.61; p<0.05	F = 12.27; p<0.05	F = 3.38; p>0.05
	Post-exercise	16.64 ± 0.73^#$^	18.25 ± 0.70^$^	15.87 ± 0.69* ^$^	15.92 ± 0.57* ^$^	14.99 ± 0.58* ^#^	14.61 ± 0.64*						

Values are expressed as mean ± SD.

* Significantly different from pre-Ramadan value for the same time-of-day.

^#^ Significantly different from 17:00 h value for the same period.

^$^ Significantly different from rest value for the same time-of-day and the same period.

TC: total cholesterol; TG: triglycerides; HDL-C; high density lipoprotein; LDL-C: low density lipoprotein; Apo-AI: apolipoprotein AI; Apo-B: apolipoprotein; Lp-a: lipoprotein particles-a; hs-CRP: high-sensitive C-reactive-protein; Hcy: homocysteine.

LDL-C, Apo-B, and Lp-a showed stable values throughout the daytime BR with a significant increase during exercise only for LDL-C and Apo-B (*P* < 0.001). During RF, these parameters displayed a decrease in 17:00 h values at rest and during exercise (*P* < 0.05). At the ER, while both morning and evening Apo-B and Lp-a levels were significantly reduced (*P* < 0.01), there was a significant diurnal variation in LDL-C with higher morning values (*P* < 0.01). HDL-C and Apo-AI values increased after the exercise and showed a significant time-of-day effect with higher evening levels BR (*P* < 0.001). The rise was greater at 17:00 than 07:00 h. Moreover, levels of these parameters tended to be higher during RF (*P* < 0.01) and the time-of-day effect was suppressed by the ER with a significant decrease in resting and post-exercise evening values (*P* < 0.05).

Hcy and hs-CRP levels increased significantly during exercise (*P* < 0.01) BR with evening higher values. During the SWR, the diurnal variation of these parameters was blunted by a reduction in the evening values with respect to BR (*P* < 0.001). During ER, mean values of Hcy showed inversed diurnal pattern with greater morning values at rest and after exercise (*P* < 0.001).

## Discussion

The aim of this study was to investigate the effect of RF on the diurnal fluctuations of lipoproteins, apolipoproteins, hs-CRP, and Hcy during the YYIRT in soccer players. The results demonstrated that HDL-C and Apo-AI tended to increase, while LDL-C, Apo-B, TC, TG, Hcy and hs-CRP decreased during RF. Moreover, the diurnal pattern of these biochemical measures was modified by the ER.

### Body composition, dietary intakes, and exercise performance

The present study indicated that BM and FM decreased during ER. These findings are in agreement with previous researches [11,34] and could reflect an increased use of lipids during RF as evidenced by previous works in Tunisian subjects [4,16]. Moreover, the decrease in BM and FM in the present study’s subjects could be related to changes in the daily energy intake since dietary records revealed a decrease in the subjects’ nutritional outcome associated with a decrease in fat and cholesterol intakes. 

Concerning the YYIRT, performance improved between 07:00 and 17:00 h BR in accordance with our previous findings [27]. Moreover, the present study’s results are consistent with our group’s previous ones in which we observed that RF modifies the diurnal pattern in aerobic performance by decreasing performance in the afternoon but not in the morning hours [27].

### Biochemical Measurements

The lipid profile is an important indicator of cardiovascular health. Studies on the RF effect on blood lipids, lipoproteins, and apolipoproteins were scarce and present inconclusive results. Our results indicated that TC and TG levels increased significantly during exercise. Similar results have been shown in trained young men during submaximal exercise [17]. BR, the results indicated a diurnal fluctuation in TG and TC with higher levels observed at 17:00 h. In this context, it has been demonstrated that postprandial lipid, lipoprotein, and apolipoprotein concentrations were affected by circadian factors [21]. 

Moreover, during the SWR and ER, values of TC and TG were similar to BR in the morning, but decreased in the evening suppressing the diurnal variation observed BR. Accordingly, previous research indicated that TG and TC levels decreased or didn’t change during RF [19]. The decrease in TG and TC shown in the present study at 17:00 h during RF could reflect the beneficial effects of fasting associated to exercise on the lipid metabolism.

According to previous research [35], the present results indicated that HDL-C and Apo-AI values increased after the exercise and showed a significant time-of-day effect during BR; the rise being greater at 17:00 h. This diurnal pattern of various biochemical markers was, recently, evidenced by our group at rest and during exercise in soccer players [19,20]. Concerning the exercise’s effect on Apo-AI, it has been shown that Apo-AI may [36] or may not [37] increase after a physical exercise.

Moreover, HDL-C and Apo-AI levels tended to be higher during RF. In this context, Adlouni et al [3] suggested that the feeding behavior that occurs during RF beneficially affects serum apolipoprotein levels. In their study, Apo-AI increased by the day 29 of RF compared with the BR levels. Similarly, recent findings showed that Apo-AI levels were higher in the latter stages of RF and post-RF in trained athletes [11,12,38].

Concerning the RF effect on biochemical responses to exercise, HDL-C and Apo-AI levels increased after the exercise consistently with the findings of Bouhlel et al [17] who reported increased plasma HDL-C during a submaximal exercise during RF. Moreover, in the present work, the time-of-day effect on HDL-C and Apo-AI was suppressed by the ER with a significant decrease in the evening values at rest and during exercise. The delayed bedtime and shortened sleep during RF could reduce the amplitude of the diurnal fluctuation of performance and biochemical responses [39]. Moreover, the modified diurnal pattern of the biological markers could be explained by the reduction in evening core temperature and the modified dietary habits [39].

Alternatively, the present study’s results indicated that mean LDL-C, Apo-B, and Lp-a levels didn’t show any diurnal fluctuation at rest and during the exercise. However, other investigators identified a significant circadian variation in these parameters [22]. In this context, although Bremner et al [22] found that these markers peaked in the morning, other investigators indicated that acrophases were typically observed in the afternoon [35]. 

In the present work, significant increase during exercise was found only for LDL-C and Apo-B but not for Lp-a levels. To the best of the authors’ knowledge, it seems that there has been no study investigating the acute effects of exercise on these biomarkers.

During RF, there was a decrease in LDL-C, Apo-B as well as Lp-a values in the evening at rest and during exercise. By the ER, there was a significant diurnal variation in these parameters with higher morning values. It has been established that a given nutrient ingested at an unusual time can induce different metabolic effects [24]. Moreover, we showed that both morning and evening levels of these parameters were significantly reduced. Similarly, Adlouni et al [3] showed that Apo-B levels fluctuated during RF but were lower during ER in healthy subjects. In addition, Chennaoui et al [12] found that Apo-B concentrations tended to decrease following RF in middle distance runners. However, concerning LDL-C levels, the present study’s results did not agree with those of previous research which indicated an increase [11] or no change [13] in trained subjects. The decrease in fat and saturated fat intake observed in the present study during RF could explain these findings. The interaction between fasting and the effect of time-of-day on physical exercise during RF leads to a decrease in LDL-C, Apo-B, and Lp-a, and may beneficially affect the normal pre-RF diurnal pattern of these parameters. In fact, these biochemical markers were discriminative for cardiovascular risk. As concluded in previous research [40], it seems that beneficial changes in lipid markers are influenced not only by the timing of blood sampling and any changes in diet (calorie restriction), but also by the decrease in initial fat and body mass, and possible changes in physical activity during RF.

The present study’s findings indicated that both Hcy and hs-CRP levels showed a significant diurnal variation BR with higher evening values. The diurnal increase in post-exercise Hcy levels could be explained by the daily variation in core temperature and resting Hcy values as previously shown by Hammouda et al [19]. Concerning hs-CRP, however, studies in healthy individuals have not demonstrated any significant circadian variation [41]. The divergence between the literature results and those of the present study on hs-CRP could be explained by differences between the investigated populations concerning the training status and the mean age [19]. Moreover, the increase in Hcy and hs-CRP during the exercise indicated increased acute-phase responses and activated inflammatory process [42]. Moreover, resting and post-exercise levels of Hcy and hs-CRP decreased significantly during RF. Similarly, previous research indicated that RF positively influenced the inflammatory status of the body explained by a decrease in the IL-6 concentrations associated with decreases in both hs-CRP and Hcy [10]. However, in judo athletes, recent findings observed that although hs-CRP increased during RF, Hcy remained stable [38]. The difference between these studies may be explained by the influence of the intense judo training in the study of Chaouachi et al [38]. The decrease in inflammatory status in the present study could be explained by the reduced daily energy and fat intakes during RF. Indeed, intermittent fasting is considered as an alternative to caloric restriction regimen, which has been shown to reduce oxidative damage to lipids, protein, and DNA which implies a protective effect against oxidative stress [43]. Furthermore, the morning-to-evening difference in resting and post exercise Hcy and hs-CRP was suppressed during the SWR in comparison with BR. It seems that the beneficial effects of caloric restriction on the inflammatory status are more pronounced in the evening after an average of 13-h of daytime fasting. The decrease in evening Hcy values is maintained during ER which led to inversed diurnal pattern. However, since Vitamin A, B12, C, and E and Folate intakes showed normal values during RF, it seems that the influence of RF on daily activities and biochemical responses is more a matter of chronobiology than calorie restriction. Moreover, the modified circadian pattern of cortisol, melatonin, and core temperature, previously observed during RF [39,43] could explain the morning-to-evening difference in hs-CRP and Hcy. In this context, Bogdan et al [44] observed a shift in the onset of cortisol secretion during RF and a rise in serum cortisol levels in the afternoon, while the morning rise was apparently delayed. In addition, the circadian distribution of hepatic and myocellular enzymes (ie, gamma-glutamyltransferase, alkaline phosphatase, creatine kinase, and glutamic transaminase), indicative of muscle injury, was also altered by RF [45]. 

A limitation of the present study is that we didn’t use a control group of untrained subjects. Moreover, oral temperature was not measured after exercise to estimate its changes with exercise at the different times-of-day. Furthermore, biochemical parameters were recorded only before and after the YYIRT. Therefore, further studies should investigate a delayed blood sample to check peak values.

In summary, the present study showed that RF and exercise can be combined favorably to reduce body mass and body fat, as well as improving lipid profiles and inflammatory status in soccer players. Indeed, prolonged intermittent fasting may have positive effects on the cardiovascular risk factors (ie, Hcy, hs-CRP, Apo-AI, Apo-B, and Lp-a). Moreover, our results suggested that calorie restriction induced by RF could modify the diurnal pattern of resting and post-exercise lipoproteins, apolipoprotein AI and B, as well as hs-CRP and Hcy levels. Since many factors (ie, diet, physical activity, time-of-day) could contribute to the modified diurnal pattern of lipid profile and inflammatory markers, it would be difficult to identify each factor’s contribution. In future research, it would be of interest to standardize diet and time-of-day of biochemical measures to detect the main concomitant effects of RF and physical activity on lipid profile and cardiovascular risk markers.
